# TLR3 and TLR4 expression in healthy and diseased human endometrium

**DOI:** 10.1186/1477-7827-6-40

**Published:** 2008-09-07

**Authors:** Svenja Allhorn, Carsten Böing, Andrea A Koch, Rainer Kimmig, Isabella Gashaw

**Affiliations:** 1 Institute of Anatomy II, University of Duisburg-Essen, Hufelandstr. 55, 45147 Essen, Germany; 2 Department of Obstetrics and Gynaecology, University of Duisburg-Essen, Hufelandstr. 55, 45147 Essen, Germany

## Abstract

**Background:**

Toll-like receptors (TLRs) play an essential role in the innate immune system by initiating and directing immune response to pathogens. TLRs are expressed in the human endometrium and their regulation might be crucial for the pathogenesis of endometrial diseases.

**Methods:**

TLR3 and TLR4 expression was investigated during the menstrual cycle and in postmenopausal endometrium considering peritoneal endometriosis, hyperplasia, and endometrial adenocarcinoma specimens (grade 1 to 3). The expression studies applied quantitative RT-PCR and immunolabelling of both proteins.

**Results:**

TLR3 and TLR4 proteins were mostly localised to the glandular and luminal epithelium. In addition, TLR4 was present on endometrial dendritic cells, monocytes and macrophages. TLR3 and TLR4 mRNA levels did not show significant changes during the menstrual cycle. In patients with peritoneal endometriosis, TLR3 and TLR4 mRNA expression decreased significantly in proliferative diseased endometrium compared to controls. Interestingly, ectopic endometriotic lesions showed a significant increase of TLR3 und TLR4 mRNA expression compared to corresponding eutopic tissues, indicating a local gain of TLR expression. Endometrial hyperplasia and adenocarcinoma revealed significantly reduced receptor levels when compared with postmenopausal controls. The lowest TLR expression levels were determined in poor differentiated carcinoma (grade 3).

**Conclusion:**

Our data suggest an involvement of TLR3 and TLR4 in endometrial diseases as demonstrated by altered expression levels in endometriosis and endometrial cancer.

## Background

Toll-like receptors (TLRs) recognize specific pathogen associated molecular patterns (PAMPs) and serve an essential role in the innate immune system by initiating and directing immune response to microbial pathogens. Human TLRs comprise a large family of 10 proteins with member-specific activators and a complex downstream signalling [1]. TLRs are expressed on various immune cells but are also present on mucosal surfaces of the respiratory, gastrointestinal and urinary tract [1]. Applying different adaptor proteins such as toll-like receptor adaptor molecule 1 (TRIF, TICAM1), myeloid differentiation primary response gene 88 (MyD88), myelin and lymphocyte protein Mal, translocation associated membrane protein (TRAM) and sterile alpha and TIR motif containing (SARM), TLRs activate signalling pathways of mitogen-activated protein kinases, nuclear factor kappa-B (NFêB), signal transducers and activators of transcription (STATs) or the activator protein 1 (AP1) [1-3]. These signalling cascades result in enhanced secretion of various pro- and anti-inflammatory cytokines such as interferons, tumor necrosis factor α (TNFα) and interleukins IL4, IL8, and IL12 [1,2]. Two studies have described the expression of human TLRs in epithelial cells within the female reproductive tract [4,5]. Other than their importance for the interaction between host and pathogen, the receptors might be involved in mucosal homeostasis as described already for the intestine and colon [6]. TLR3 is implicated in the recognition of dsRNA, mRNA and viruses [1,7], whereas TLR4 is a key component of the initial injury response by reacting towards bacterial endotoxin and multiple endogenous ligands [8]. Recent studies have determined the expression pattern of TLR3 [4,9-12] and TLR4 [4,10-14] in the human endometrium, but their possible involvement in the pathogenesis of endometrial diseases associated with inflammation remains to be elucidated.

Endometriosis is a common benign gynaecological condition of reproductive aged women [reviewed in [15]]. The disease is characterised by endometrial tissue fragments outside the uterine cavity and is associated with pelvic pain, dysmenorrhoea, and infertility. Since aetiology and pathogenesis remain uncertain, different theories are discussed including altered immune function. The deregulation of immune response in endometriosis is characterised by increased number of activated macrophages and their secreted products, such as growth factors, cytokines, and angiogenic factors [16,17]. Young et al. reported an increase in interleukin-8 (IL-8) production after stimulating TLR3 and TLR4 in endometrial cell lines with appropriate ligands [12]. IL-8 is a chemotactic activating cytokine for leukocytes and it has been hypothesized to play a role in the growth and maintenance of ectopic endometrial tissue [18]. Recent studies consider endometriosis as a process of sterile inflammation in the pelvis, which is accompanied by elevated levels of inflammatory key regulators such as TNFα [19] or NF-κβ [20]. Both are known downstream targets of TLRs.

Endometrial carcinoma is the most common gynaecological malignancy in Europe and North America affecting mainly postmenopausal women [21]. In endometrial tumorigenesis, two different types are characterised: the estrogen-related adenocarcinoma (endometrioid type) and the non-endometrioid type such as papillary serous and clear cell carcinoma [21]. Adenocarcinoma accounts for seventy percent of endometrial cancer and is mostly preceded by premalignant changes like endometrial hyperplasia [21]. The majority of adenocarcinoma expresses steroid receptors and occur in women with risk factors associated with an imbalance of estrogen and progesterone. However, inflammation with production of pro-inflammatory cytokines such as TNFα is known to play an important role in cancer development [22]. In endometrial hyperplasia and adenocarcinoma the expression of NFκB and TFNα has been demonstrated [23] indicating that the production of pro-inflammatory cytokines seem to play a role in endometrial tumorigenesis.

The present study describes the expression pattern of TLR3 and TLR4 mRNA and proteins in healthy endometrium across the menstrual cycle and in postmenopausal tissue. To assess the possible involvement of these toll-like receptors in endometrial pathologies, their expression pattern was also examined in endometriosis and in adenocarcinoma specimens.

## Methods

### Endometrial tissues

Endometrial tissues were obtained from 55 women with regular menstrual cycles (mean 28 ± 2.2 days) who were undergoing gynaecological procedures for benign conditions at the Department of Gynaecology, University Hospital Essen (table 1). In this cohort, 20 women have been diagnosed with endometriosis. Menstrual effluents were collected from women without proven endometriosis during first three days of menstrual bleeding as described elsewhere [24].

**Table 1:** Patients' characteristics according to diagnosis at time of surgery

	**n**	**Age, mean**	**Age, SD**	**Indications for surgery**
Premenopausal, controls (proliferative & secretory)	27	37	8.6	fibroids (n = 7), non endometriotic ovarian cyst (n = 2), infertility (n = 5), dysmenorrhoe (n = 11), pelvic pain (n = 1), uterine prolapse (n = 1)
Premenopausal, non-endometriotic, menstrual	8	39	10.7	no surgery
Premenopausal, endometriotic	20	34	6.8	endometriosis (n = 11), ovarian cyst (n = 2), infertility (n = 1), dysmenorrhoe (n = 6*)
Postmenopausal, controls	8	68	10.2	fibroids (n = 6), uterine prolapse (n = 2)
Postmenopausal, hyperplasia	10	64	13	abnormal endometrial thickness, supposed carcinoma
Postmenopausal, endometrial carcinoma	16	67	12.8	abnormal endometrial thickness, endometrial carcinoma

n = number of patients, * not including patients with known endometriosis before surgery

The menstrual cycle phase was characterised by morphologic evaluation following the criteria of Noyes et al. [25]: proliferative (P, controls: n = 16, endometriotic: n = 13), secretory (S, controls: n = 11, endometriotic: n = 3) and menstrual (M, n = 8) phase. Additionally, four proliferative corresponding ectopic lesions were included, obtained from the above-characterised cohort. In the premenopausal group, patient age ranged from 19 to 52 years (median: 38, detailed data in table 1).

Postmenopausal endometrium was obtained from 34 women including 10 with endometrial hyperplasia and another 16 with endometrial carcinoma (table 1). The remaining 8 patients did not have any endometrial abnormalities and were used as the control group. Patients were considered postmenopausal if they have been in menopause for at least one year. Endometrial adenocarcinoma specimens were classified based on the post-operative histopathologic WHO guidelines [26] as follow: grade 1 (G1, well differentiated, n = 5), G2 (moderately differentiated, n = 6), G3 (undifferentiated, n = 5). In the postmenopausal group, patient age ranged from 37 to 86 years (median: 66).

None of the women included in the studies had received any hormonal treatments for at least three months preceding biopsy and routinely analyzed laboratory parameters from blood samples were physiologically analogous to the patient's age. Considering the leukocyte content and level of C-reactive protein, no systemic inflammation was diagnosed at the time of surgery. Volunteers donating menstrual effluents were healthy and without diagnosed infections.

Institutional ethical approval was granted for all subjects, and all women provided written informed consent.

All biopsies were transferred into a buffered saline solution directly after surgery and stored in this buffer for maximal two hours until further use. A portion of the biopsy specimen was fixed in 4% formalin and embedded in paraffin for histology and immunohistochemistry, the remainder was flash-frozen in liquid N_2 _for RNA extraction.

### Quantitative real-time PCR

Isolation of total RNA from endometrial tissue and reverse transcription into cDNA were carried out applying standard methods as described previously [24]. Following a DNase digest and reverse transcription, quantitative real-time PCR (qPCR) reactions were performed in triplicates using an ABI Prism 7300 Sequence Detector (Applied Biosystems, Weiterstadt, Germany) in a total volume of 20 μl containing 40 ng cDNA, 3.75 pmol gene-specific primers (table 2) and SYBR Green reagent (Applied Biosystems) with ROX dye as passive control for signal intensity. The thermal cycle profile was 10 sec at 95°C, followed by 45 cycles of 5 sec at 95°C and 35 sec at 60°C. Melting curve analysis allowed determination of the specificity of the PCR fragments. All melting curves yielded one peak per PCR product.

**Table 2:** Oligonucleotide primers used for the quantitative real time PCR.

**Gene (GenBank No.)**	**Forward primers (position)**	**Reverse primers (position)**
TLR3 (NM_003265)	5'-GTATTGCCTGGTTTGTTAATTGG (2059–2082)	5'-AAGAGTTCAAAGGGGGCACT (2215–2194)
TLR4 (NM_138557)	5'-AAGCCGAAAGGTGATTGTTG (2187–2206)	5'-CTGAGCAGGGTCTTCTCCAC (2339–2320)
ACTB (NM_001101)	5'-ACCAACTGGGACGACATGGA (302–322)	5'-CCAGAGGCGTACAGGGATAG (510–491)

All primers were designed using the Primer3 software.

To determine the copy number of PCR fragments, serially diluted, gene specific standard cDNAs generated from amplicons of TLR3, TLR4 and β-actin (ACTB) were used. Applying thermal block cyclers and ethidium bromide gel electrophoresis, standard PCRs were conducted. Each gene-specific PCR resulted in one distinct band of the appropriate length. The amplicons were purified by using a Qiagen kit and cDNA concentration was measured photometrically. For each gene, five different dilutions of standard cDNA were used in real time PCR. Threshold cycles for TLR3 signals were between 26 and 38 and for TLR4 between 25 and 36, respectively. Because of the diversity in the RNA quality, each individual sample was normalized to its ACTB mRNA content as an internal standard. These relative values were used for statistics.

### Immunohistochemistry

Paraffin-embedded specimens were sectioned at 7 μm, rehydrated and microwaved in 0.01 M sodium citrate buffer, pH 6.0, for 10 min for antigen retrieval. Immunostainings were performed on paraffin sections applying the diaminobenzidine staining method with the VECTASTAIN Elite ABC kit (Vector Laboratories, Burlingame, CA) according to the manufacturer's protocol. Endogenous peroxidase activity was quenched with 0.3% H2O2 in methanol for 10 minutes and washed in buffered saline solution (PBS). Unspecific binding of the first antibody was blocked by 30-minute incubation step in PBS containing 0.15% normal horse serum. Slides were incubated in a humidified chamber overnight at 4°C with the monoclonal mouse-anti-human antibodies against TLR3 [27] and TLR4 [HTA125, [28]] at 20 μg/ml and 100 μg/ml, respectively (Acris Antibodies, Hiddenhausen, Germany). Control samples were carried out by omitting the primary antibody. All sections were counterstained with haematoxylin and documented by using a Zeiss Axiophot microscope (Zeiss, Jena, Germany) with a Nikon DS-U1 camera and the LUCIA Image Analysis software (Nikon, Tokyo, Japan).

### Immunofluorescent staining

Frozen tissues were sectioned at 7 μm and fixed in 70% ethanol. Unspecific binding of the first antibody was blocked by a 30 min incubation step in 5% BSA/PBS. The TLR4 antibody was incubated as described above and was detected using Alexa Fluor 488-conjugated anti-mouse antibody (3.3 μg/ml, MoBiTec, Goettingen, Germany). Sections were fixed in formalin (4%) for two minutes and then washed in PBS. The incubations with CD14 (10 μg/ml, mouse anti-human, BioLegend, San Diego, CA) or CD163 (10 μg/ml, mouse anti-human, HyCult Biotechnology, Uden, The Netherlands) occurred at room temperature for 60 min. CD14 antigen is expressed on on monocytes/macrophages, acting as a dendritic cells precursor [29]. CD163 is a member of the scavenger receptor cystein-rich family class B and is expressed on most subpopulations of mature tissue macrophages [30]. CD163 is highly abundant in human placenta [31] and is present in shed menstrual endometrium [24]. The secondary, goat anti-mouse antibody was Cy3-conjugated (2.5 μg/ml, Dianova, Munich, Germany) and was applied to the specimens for another 60 minutes. Nuclei were identified by 4',6'-diamidino-2-phenylindole staining (DAPI, Sigma, Munich, Germany) using 0.1 μg/ml DAPI in methanol for 15 min at 37°C. Negative controls were performed by omitting the primary antibody and were used to adjust the background fluorescence.

After mounting with Mowiol (Sigma), confocal microscopy was performed using a Zeiss Axiovert 100 microscope and LSM 510 system (Zeiss, Jena, Germany). TLR4 was detected at 488 nm, CD14 as well as CD163 at 543 nm, and DAPI at 366 nm, respectively.

### Statistical analysis

Exploratory data analyses, Kruskal-Wallis test for group comparisons, as well as the Mann-Whitney *U *test for nonparametric independent two-group comparisons were performed with the program SPSS 14 for Windows (SPSS Inc., Chicago, IL). Differences with *P *< 0.05 were regarded as statistically significant, *P *< 0.01 as highly statistically significant. Values of mRNA quantification are given as mean ± standard deviation (SD).

## Results

### TLR3 and TLR4 expression is deregulated in peritoneal endometriosis

**Figure 1:**
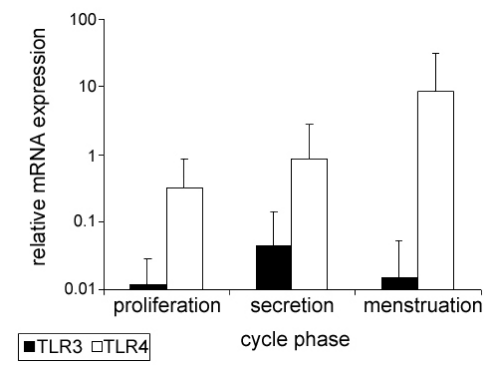
**TLR3 and TLR4 transcript are expressed in endometrium during the menstrual cycle**. Columns indicate mean TLR3 and TLR4 mRNA quantities from endometrium in proliferative (n = 16), secretory (n = 11) and menstrual phase (n = 8) run in triplicates. The y-axis is scaled logarithmically; error bars represent the standard deviation of the mean.

Both receptors were expressed in all endometrial biopsies with the averaged TLR4 mRNA levels being higher (20-fold) than TLR3 (figure 1). This difference was the greatest in the shed menstrual endometrium, where TLR4 transcripts were 564-fold higher than those for TLR3. The relative abundance of both transcripts did not vary throughout the menstrual cycle (figure 1).

**Figure 2:**
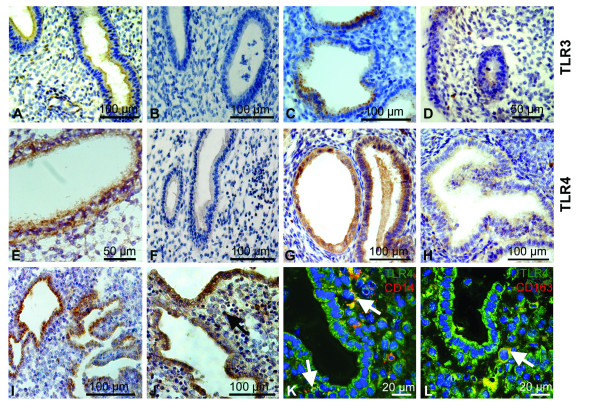
**TLR3 and TLR4 protein is localised to endometrial cells during the menstrual cycle**. TLR3 protein staining in healthy late proliferative (LP) tissue was high in luminal and glandular tissue **(A**, brown precipitate) and lower in LP endometriotic tissue **(B)**. Late secretory (LS) endometrium showed highly expressed TLR3 in the epithelium **(C)**, but weakly in endometriosis **(D)**. Intense staining of TLR4 proteins was shown in mid proliferative (MP) tissue **(E)**. In late proliferative phase of endometriosis, TLR4 proteins were comparably lower **(F)**. TLR4 protein was high in mid secretory (MS) normal endometrium **(G)**, whereas it was decreased in endometriotic MS tissue **(H)**. During the menstrual phase, both TLR3 **(I) **and TLR4 **(J) **were highly expressed. Co-immunostaining for TLR4 (green), CD14 (**K**, red) and CD163 (**L**, red) demonstrated that TLR4 proteins were expressed by CD14 positive dendritic cells and monocytes (**K**, yellow) and by CD163 positive macrophages (**L**, yellow). Localisation of TLR4 to immune cells is marked by a black arrow **(J) **and by white arrows **(K, L)**.

TLR3 and TLR4 proteins were expressed mainly in the luminal and glandular epithelium (figure 2). Interestingly, the glands presented a heterogeneous immune staining for TLR3 (figure 2C, I). Indeed, the TLR3 receptor was found to be locally expressed in a subset of epithelial cells within one gland. In addition, we report the expression of TLR4 protein on immune cells such as monocytes and macrophages, in menstrual phase samples (figure 2J). Co-immunostainings on menstrual effluents confirmed that CD14 positive dendritic cells and monocytes (figure 2K) as well as CD163 positive resident macrophages (figure 2L) expressed TLR4 protein.

**Figure 3:**
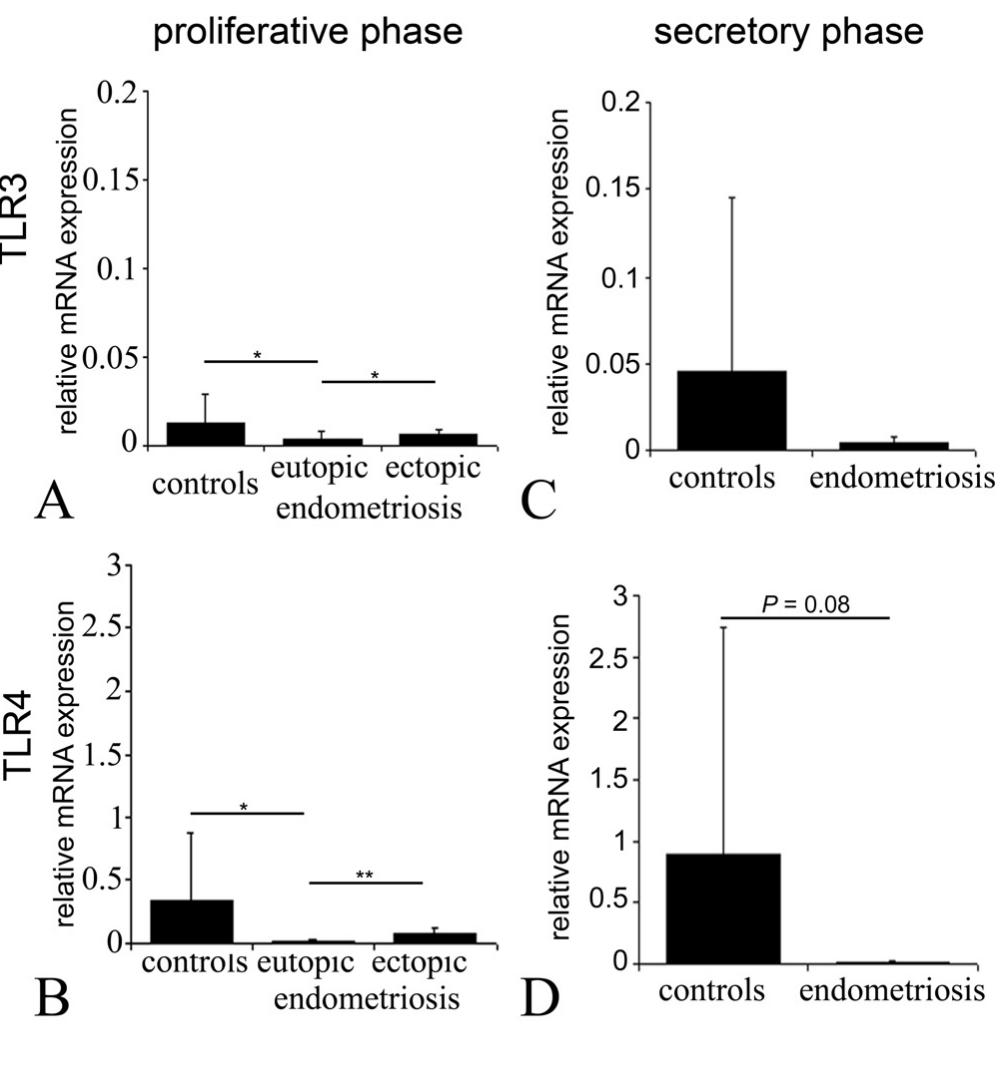
**TLR3 and TLR4 mRNA expression is regulated in endometriosis**. The expression of TLR3 **(A, C) **and TLR4 mRNA **(B, D) **in endometrium during proliferative (n = 13, run in triplicates, **A, B) **and secretory phase (n = 3, **C, D) **was decreased in eutopic endometriotic endometrium when compared to controls. In addition, four proliferative corresponding lesions were evaluated **(A, B) **showing a local upregulation of both receptors on ectopic sites. Columns represent the mean ratio of TLR copy number to ACTB copy number. Error bars represent the standard deviation of the mean. * P < 0.05; ** P < 0.01.

In endometriosis, we can observe a significant decrease in TLR3 and TLR4 mRNA levels in eutopic tissues collected during the proliferative phase, when compared to controls (*P *< 0.05; figure 3A–B). Interestingly, endometriotic lesions in proliferative phase showed a significant increase of TLR3 mRNA expression (*P *< 0.05) when compared with the corresponding eutopic endometrium (figure 3A). For the TLR4 transcript, a 6-fold increase was observed in the endometriotic lesions in comparison with the diseased eutopic endometrium (*P *< 0.01; figure 3B). In the endometrial tissues collected during the secretory phase, the TLR4 mRNA level tended to be lower in eutopic endometrium than in controls (*P *= 0.08; figure 3D).

**Figure 4:**
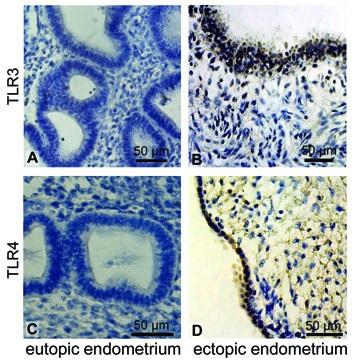
**TLR3 and TLR4 protein is locally induced in endometriotic lesions**. No specific TLR3 protein staining was seen in eutopic endometriotic tissue **(A) **whereas a high glandular localisation of the protein was detected in a gland of an ectopic endometriotic lesion from the same patient **(B)**. Similarly, TLR4 was not detectable in eutopic endometrium **(C) **but present in glandular epithelium of ectopic endometrium from the same women **(D)**.

Immunostaining analyses confirmed these findings at the protein level. In endometriosis, eutopic tissues revealed weaker staining for TLR3 and TLR4 proteins (figure 2B, D, F, H) when compared to controls (figure 2A, C, E, G). Figure 4 exemplary presents the expression of TLR3 and TLR4 protein in eutopic compared to ectopic endometrium from the same patient. The TLR3 (Fig. 4A) and TLR4 (Fig. 4C) immunostaining in diseased eutopic endometrium was barely detectable, whereas corresponding lesion from the uterosacral ligament showed an intense staining in the glandular epithelium for both proteins (Fig. 4B and 4D). Concerning protein localization, we found TLR3 and TLR4 proteins in glandular and epithelial cells of endometriosis patients.

### TLR3 and TLR4 are expressed in postmenopausal endometrium and regulated endometrial adenocarcinoma

**Figure 5:**
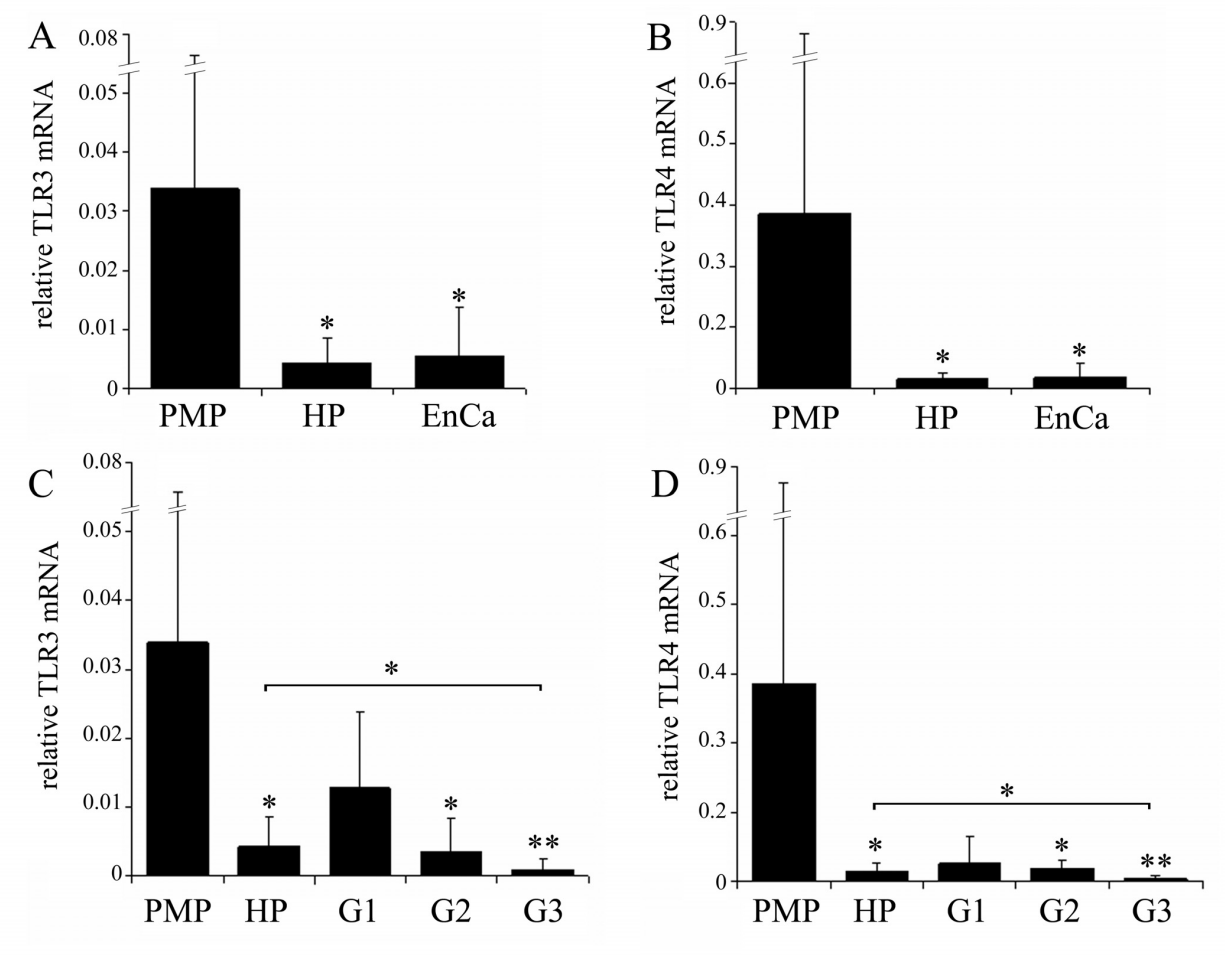
**TLR3 and TLR4 mRNA expression is decreased in endometrial adenocarcinoma**. **(A-B) **Columns indicate mean TLR3 (A) and TLR4 (B) mRNA levels from postmenopausal patients (PMP, n = 8), and those diagnosed with endometrial hyperplasia (HP, n = 10) and endometrial carcinoma (EnCa, n = 16). **(C-D) **TLR3 (C) and TLR4 (D) mRNA expression in different carcinoma grades compared to postmenopausal controls and hyperplastic endometrium: G1 (n = 5), G2 (n = 6) and G3 (n = 5). Error bars represent the standard deviation of the mean. * P < 0.05; ** P < 0.01.

TLR3 and TLR4 mRNA abundance in healthy postmenopausal tissues is similar to those found during the menstrual cycle. In postmenopausal controls, TLR4 mRNA levels were higher than those for TLR3 (*P *< 0.05, figure 5). TLR3 and TLR4 mRNA expression varied significantly between control, hyperplasia and endometrial adenocarcinoma samples (Kruskal-Wallis test, *P *< 0.01). For both receptors, we observe a significant decrease in mRNA abundance in endometrial hyperplasia and adenocarcinoma samples, when compared to postmenopausal endometrium (*P *< 0.05; figure 5A–B). In undifferentiated G3 carcinoma, TLR3 and TLR4 mRNA levels were significantly lower than in postmenopausal controls (*P *< 0.01) and in hyperplasic endometrial tissues (*P *< 0.05, figure 5C–D).

**Figure 6:**
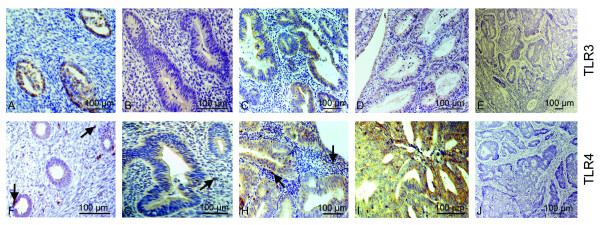
**TLR3 and TLR4 proteins are present in postmenopausal endometrium**. Localisation of TLR3 in normal postmenopausal endometrium **(A)**, endometrial hyperplasia **(B)**, endometrial adenocarcinoma grade G1 **(C)**, G2 **(D) **and G3 **(E**). Localisation of TLR4 protein in normal postmenopausal endometrium **(F)**, endometrial hyperplasia **(G)**, endometrial adenocarcinoma grade G1 **(H)**, G2 **(I) **and G3(**J**). All stained sections indicated epithelium as the preferred localisation of TLR3 and TLR4 proteins. TLR4 protein was additionally present in immune cells (arrows).

**Figure 7:**
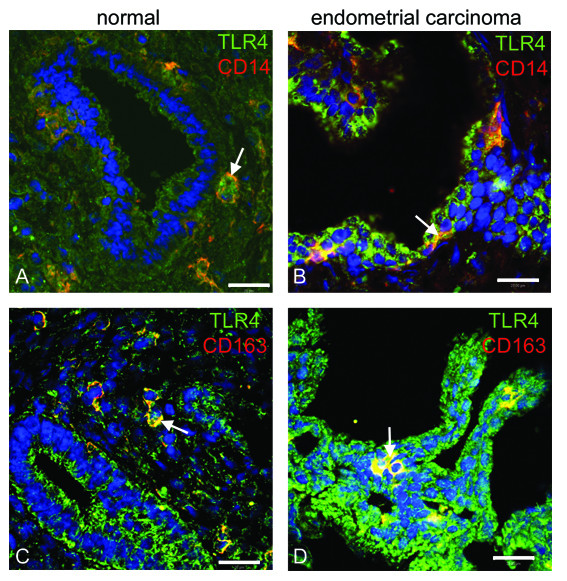
**TLR4 is localised to immune cells of postmenopausal endometrium**. Co-Immunostaining of TLR4 with CD14 and CD163 in healthy endometrium **(A, B) **and in adenocarcinoma **(C, D)**. TLR4 proteins were expressed by CD14 positive dendritic cells and monocytes **(A, C) **and by CD163 positive macrophages **(B, D)**. Arrows indicate the co-localisation of TLR4 with the immune cells. Scale bar = 20 μm.

TLR3 and TLR4 proteins in hyperplasia and endometrial carcinoma were mostly localized to the luminal and glandular epithelium (figure 6). Additionally, we demonstrate a discontinuous staining for TLR3 protein within epithelial glands of G1 carcinoma (figure 6C), comparable to the findings in secretory and menstrual phase of premenopausal women (figure 2C, I). In undifferentiated G3 carcinoma, staining for TLR3 (figure 6E) and TLR4 (figure 6J) was not detectable, strengthening our findings of low TLR3 and TLR4 mRNA abundance in G3 carcinoma (figure 5B). In accordance with staining patterns obtained during the menstrual phase (figure 2J), we were able to find TLR4 protein localized on immune cells (figure 6F, G, H, I). We performed co- immunostainings on controls and on malignant endometrial tissues (G2 carcinoma) with antibodies for CD14 and CD163 (figure 7). TLR4 protein was expressed on CD14 positive dendritic cells, and monocytes (figure 7A, C), as well as on CD163 positive macrophages (figure 7B, D).

## Discussion

In the current study, we report that toll-like receptor 3 and 4 expression is modulated in pathogenic alterations of the endometrium. We also found higher TLR4 expression levels in endometrial samples throughout the menstrual cycle and in postmenopausal biopsies, when compared to those for TLR3. In most tissues including gut, gonads and placenta, TLR3 is greater expressed than TLR4 mRNA [32]. TLR3 recognizes RNA and viruses, whereas TLR4 mediates the response to bacterial endotoxins and is activated due to sterile inflammation [33,34]. Thus, the predominant expression of TLR4, observed in uterine tissues, might reflect the occurrence of sterile inflammation during the menstrual cycle. Moreover, ascending bacterial pathogens could contribute to the TLR4 dominance in the uterus.

In agreement with earlier reports [4,12], both investigated TLRs were mainly localized in the endometrial epithelium, the site of primary immune response in the uterus. In addition, we were able to detect TLR4 protein on endometrial CD14 and CD163 positive immune cells. We found CD14 mainly expressed within the epithelial layer, only a sporadic number of CD14 positive cells was detected in stromal compartment, probably representing the population of monocytes. A recent study performed on bovine endometrial cells, co-localised TLR4 transcripts with CD14 mRNA and protein to stromal and epithelial cells [35]. CD14 is a known accessory molecule for TLR4 and conducts a downstream signalling cascade via MyD88 [3]. In agreement with Pioli et al., who detected TLR4, CD14 and MyD88 transcripts in human endometrium [14], we were able to co-localize TLR4 with CD14 proteins suggesting the presence of both interacting receptors CD14 and TLR4 in the endometrial cells.

For the first time, we present endometrial effluents expressing high levels of TLR3 and TLR4 proteins. Since the period of menstruation is accompanied by an increased risk of infections due to ascending microorganisms [36], we believe that the increased expression of toll-like receptors may be one of the defense mechanisms used by the uterus. Previous studies reported that toll-like receptors are also implicated in epithelial repair as described for intestinal [6] and alveolar epithelial cells [37]. In damaged tissue, necrosis induced inflammation is thought to trigger danger signals, leading to tissue repair response through TLRs [6]. Since repair processes occur every month in the uterus of premenopausal women, we believe that the interaction between hyaluronan and TLR4 might promote the endometrial repair. Hyaluronan is released by necrotic cells, interacts with TLR4 and activates CD44 mediated signalling [38]. In the endometrium, deposition of hyaluronan has been described in stromal compartment [39]. Moreover, hyaluronan has been reported to be involved in attachment of endometrial cells to the mesothelium as a very early step of endometriosis [40]. Further investigations of hyaluronan-TLR4 signalling in healthy and diseased endometrium would be of interest to gain insight into the functional role of TLR4 expression in the uterus.

Endometriosis causes chronic inflammatory conditions in the pelvic cavity and in the uterus. This disorder is discussed to be accompanied by an activation of the Th2 type of immune response and a shift from Th1 towards Th2 cytokine production [41]. Interestingly, Th2 cytokines were shown to play an important role in balancing TLR signalling in human intestinal epithelial cells by mediating downregulation of TLR3 and TLR4 expression and function [42]. This could also be the case in the diseased eutopic endometrium, where decreased TLR levels were found. It remains to be fully elucidated, if deregulation of TLR expression is involved in the pathogenesis of endometriosis or if altered TLR expression patterns are a consequence resulting from the presence of endometriotic lesions. We could recently show that uterine gene expression patterns are altered due to the existence of ectopic lesions in a non-human primate model for endometriosis [43,44]. Since implantation is a process accompanied by an inflammatory event, an impaired fertility observed in endometriotic women could be one consequence [45]. Continued studies are needed to determine the role of TLR function in diseased endometrium, which could be a promising path towards a better understanding of the pathogenesis of this disease.

Interestingly, we found a local upregulation of both TLRs in peritoneal endometriotic lesions when compared to eutopic endometriosis from the same patients. A recent study presented a local upregulation of CD14 and CD163 in ovarian endometriotic lesions [46]. However, we observe locally gained TLR4 expression in epithelial cells as demonstrated in immunohistochemical stainings. We propose that the sterile inflammation process, which occurs in the pelvic cavity upon endometriosis, is able to enhance the epithelial TLR4 expression and thus activate the known downstream signalling cascade. One of the potentially activated TLR-downstream molecules is NF-κB, which was recently found as constitutively elevated in endometriotic lesions [20]. It is established that the activation of NF-κB is linked to proliferation, angiogenesis and enhanced production of inflammatory cytokines on ectopic sites [17]. Hence, the TLR-NF-κB cascade might contribute to the chronic persistence of endometriotic lesions.

In endometrial adenocarcinoma, expression levels of the downstream molecules TNFα and NF-κB were decreased in G2 and G3 but not in the well-differentiated grade 1 carcinoma [47]. Although the evidence is lacking, the almost negligible levels of both toll-like receptors in G3 endometrial adenocarcinoma may reflect lowered differentiation and possibly indicates poor prognosis.

Both endometriosis and endometrial adenocarcinoma are estrogen dependent diseases. In both conditions, TLR3 and TLR4 were significantly decreased in diseased endometrium when compared to age matched controls. However, it is known that estrogen did not influence the expression of either TLR3 [48] nor TLR4 [49,50] in epithelial cells of endometrium [48], retina [50] and in macrophages [49]. Thus, additional factors are required to decrease TLR-expression in endometriotic endometrium and in endometrial carcinoma.

Besides excessive estrogen, genetic predisposition presents one of the risk factors associated with the development of endometrial adenocarcinoma. In our study, we observed high inter-individual differences in TLR expression as mirrored by high standard deviations. The results implicate a possible impact of polymorphisms on mRNA expression in physiologic and pathologic endometrium. Recently, a functional polymorphism of the TLR4 gene, associated with impaired TLR signalling, was considered as a significant risk factor for gastric carcinoma [51]. Another single nucleotide polymorphism in 3'-untranslated region of the same gene has been associated with increased risk for prostate carcinoma [52]. It remains to be fully elucidated, if genetic polymorphisms in genes encoding for toll-like receptors might promote endometrial carcinogenesis.

## Conclusion

Our data suggest an involvement of TLR3 and TLR4 in endometrial diseases as we demonstrated altered expression levels for both receptors in endometriosis and endometrial adenocarcinoma. Healthy and differentiated endometrium seems to require an adequate TLR3 and TLR4 expression. Further studies are necessary to investigate the potential function of both receptors in endometrial diseases.

## Competing interests

The authors declare that they have no competing interests.

## Authors' contributions

SA processed tissue samples, established the TLR-assays, carried out the expression analyses, analyzed data, and drafted the manuscript. CB participated in the design of the study, collected patients' tissues, and was involved in the analyses of data. AAK was involved in tissue processing and expression analyses. RK participated in the design and interpretation of the study. IG conceived the study, participated in its design, coordination, and analysis, and helped to draft the manuscript. All authors have read and approved the final manuscript.
